# Lifetime cost-effectiveness simulation of once-weekly exenatide in type 2 diabetes: A cost-utility analysis based on the EXSCEL trial

**DOI:** 10.1016/j.diabres.2021.109152

**Published:** 2022-01

**Authors:** Frauke Becker, Helen A. Dakin, Shelby D. Reed, Yanhong Li, José Leal, Stephanie M. Gustavson, Eric Wittbrodt, Adrian F. Hernandez, Alastair M. Gray, Rury R. Holman

**Affiliations:** a Health Economics Research Centre, University of Oxford, Oxford, UK; b Duke Clinical Research Institute, Duke University School of Medicine, Durham, NC, USA; c AstraZeneca Research and Development, Gaithersburg, MD, USA; d Diabetes Trials Unit, University of Oxford, Oxford, UK

**Keywords:** CVD, Cardiovascular disease, EQ-5D, EuroQol 5-Dimension, EQW, Once-weekly exenatide, EXSCEL, Exenatide Study of Cardiovascular Event Lowering, GLP-1 RA, Glucagon-like peptide-1 receptor agonist, ICER, Incremental cost-effectiveness ratio, QALY, Quality-adjusted life-year, UKPDS-OM2, UK Prospective Diabetes Study Outcomes Model version 2, Costs, Cost-effectiveness, Economic evaluation, Exenatide, Glucagon-like peptide-1 receptor agonist, Type 2 diabetes mellitus

## Abstract

**Aims:**

The Exenatide Study of Cardiovascular Event Lowering (EXSCEL) trial assessed once-weekly exenatide (EQW) vs. placebo, added to usual care in 14,752 patients with type 2 diabetes mellitus (Clinicaltrials.gov: NCT01144338). We assessed the lifetime cost-effectiveness of adding EQW vs. usual care alone from a healthcare perspective.

**Methods:**

Medical resource use and EQ-5D utilities were collected throughout the study. Within-trial results were extrapolated to a lifetime horizon using the UK Prospective Diabetes Study Outcomes Model version 2 (UKPDS-OM2), predicting predict cardiovascular and microvascular events. Cost-effectiveness was evaluated separately for US and UK settings, with outcomes measured in quality-adjusted life-years (QALYs).

**Results:**

EQW plus usual care gained 0.162 QALYs at an additional cost of $41,545/patient, compared with usual care in a US setting. The incremental cost-effectiveness ratio (ICER) was $259,223/QALY. In a UK setting, the QALY gain was 0.151 at an additional cost of £6357: an ICER of £42,589/QALY. Sensitivity analyses ranged between $34,369–$269,571 and £3430–£46,560 per QALY gained.

**Conclusions:**

In a lifetime extrapolation, adding EQW to usual care increased QALYs and costs compared with usual care alone. The base-case ICERs exceeded the commonly-cited cost-effectiveness thresholds of $100,000/QALY and £20,000/QALY. However, ICERs were considerably lower in some subgroups, and in sensitivity analyses.

## 1 Introduction

The prevalence of type 2 diabetes and associated health problems continue to rise, imposing significant burden on healthcare systems.[1] Individuals with type 2 diabetes are at higher risk of long-term complications and death from any cause,[2] and experience lower quality of life due to diabetes-related complications such as cardiovascular disease (CVD), stroke, heart failure, renal failure, amputation, and blindness.[3] While improved glycemic control has been associated with improved microvascular outcomes,[4] effects on macrovascular outcomes are more modest.[5] Exenatide, a glucagon-like peptide-1 receptor agonist (GLP-1 RA), has been shown to reduce glucose levels in individuals with type 2 diabetes, along with modest reductions in body weight, blood pressure, and lipid levels.[6]

The Exenatide Study of Cardiovascular Event Lowering (EXSCEL) assessed the effect of once-weekly exenatide (EQW, Bydureon) 2 mg vs. placebo when added to usual care in 14,752 patients with type 2 diabetes with or without previous CVD.[7] This pragmatic, randomized, double-blind, placebo-controlled, event-driven trial found that patients randomized to EQW had a numerically lower incidence of major adverse cardiovascular events, and a nominally significant reduction in all-cause mortality.[8] Patients in EXSCEL receiving EQW were found to experience fewer inpatient days during the trial period, while health-related quality of life and the number of outpatient visits to all providers were similar across treatment groups.[9] Total within-trial costs exclusive of study medication were lower in the EQW vs. the placebo group (p ≤ 0.01), but significantly higher (p < 0.01) when the estimated cost of branded EQW was included. Previous trials have compared EQW to an active comparator, such as other GLP-1 RAs, but had a smaller sample size or a shorter follow-up than EXSCEL.[10], [11], [12], [13] Our analysis combines the clinical data from the trial with the data on within-trial costs and quality of life and extrapolates the events and risk factors beyond the end of the trial to evaluate lifetime cost-effectiveness.

We extrapolated the results of the EXSCEL trial to estimate the lifetime cost-effectiveness of adding branded EQW to usual care, compared with usual care alone, from a healthcare perspective in both US and UK settings.

## 2 Materials and methods

We conducted a cost-utility analysis based on data from the EXSCEL trial, the only study to have assessed long-term outcomes of EQW added to usual care compared to usual care alone. Resource use and EuroQol 5-Dimension (EQ-5D) utility data were prospectively collected for each trial participant during trial follow-up of up to 6.7 years, and used to calculate within-trial costs and quality-adjusted life-years (QALYs) using the methods described elsewhere.[9]

We used the UK Prospective Diabetes Study Outcomes Model version 2 (UKPDS-OM2)[14] to extrapolate outcomes for individual trial participants from end of follow-up until death. The UKPDS-OM2 is a validated and widely used computer simulation model based on patient data from the UKPDS trial.[14] It predicts the occurrence of eight diabetes-related complications over a lifetime horizon and allows lifetime healthcare costs and quality-adjusted life survival to be estimated for individual participants. To extrapolate costs and outcomes beyond the trial period, we entered data on cardiovascular risk factors (*e.g.* age, lipoproteins, blood pressure, glycated hemoglobin and prior clinical events) for individual trial participants at the end of study follow-up into the UKPDS-OM2.

In the base-case analysis, all risk factors other than age, duration of diabetes and diabetes-related complications (which are updated annually within the UKPDS-OM2) were held constant throughout the extrapolation period at the levels observed at the end of the trial. Similarly, patients were assumed to continue to receive EQW (where appropriate) and any concomitant medication at the same level of compliance observed in the last year of the trial until death or development of renal failure (at which point participants were assumed to discontinue EQW and accrue the same cost as participants receiving standard care); see the Additional Methods and Tables S1-S4 in the online Supporting Information for details.

The reference year for costs was 2017 for the US analysis and 2016 for the UK analysis. Costs, QALYs, and life-years were discounted at 3% per annum in the US analysis and 3.5% in the UK analysis.[15], [16] The US base case applied a 23.1% discount on the wholesale price of branded EQW to capture the mean discount on the average manufacturer’s price of brand-name medications for Medicaid.[9]

Quality of life at each within-trial time point was measured using either the EQ-5D-5L or the EQ-5D-3L; we used value sets for the US[17] and the UK,[18] after converting EQ-5D-5L responses to EQ-5D-3L using the crosswalk algorithm.[19] Following the area-under-the-curve approach, we estimated QALYs over each participant’s trial follow-up period by assuming linear changes in utility between observed time points. These were then combined with each participant’s predicted quality-adjusted survival after follow-up, as estimated by the UKPDS-OM2.

Missing data on baseline EQ-5D utilities, height, and weight were imputed using the mean value (by sex). Missing smoking status was imputed using the country mode. Multiple imputations were performed using chained equations models and STATA’s ‘mi impute’ command to impute missing post-baseline EQ-5D utilities and risk factor values separately (STATA version 14.2); see online Supporting Information for details. We used 28 imputed data sets for missing EQ-5D values as 28% of patients had missing data.[20] We averaged across all imputed risk factor values and combined these with the observed risk factor data as inputs for the UKPDS-OM2.

Uncertainty around costs and QALYs during the trial and in the extrapolated period was taken into account as described in the Online Supporting Information. In brief, 800 bootstrapped estimates of within-trial costs and QALYs were added to 800 bootstrapped estimates of costs and QALYs in the extrapolated period to give a measure of the uncertainty around total lifetime costs and QALYs. Estimates of within-trial QALYs took account of uncertainty around imputed post-baseline utility values and were adjusted for baseline EQ-5D utility to avoid any bias associated with baseline imbalance.[21] Confidence intervals around incremental cost-effectiveness ratios (ICERs) were based on the 2.5th and 97.5th percentiles across all bootstraps of all imputed datasets. Cost-effectiveness acceptability curves were constructed by estimating the proportions of all bootstraps across all imputed datasets that were cost-effective across a range of ceiling ratios representing society’s willingness to pay for a QALY.

To assess the robustness and generalizability of the results, we conducted 14 (US) or 15 (UK) sensitivity analyses, which varied assumptions and methods such as those concerning risk factor progression, missing data, utility values (*e.g.* Lung utilities in the US setting[22]), discount rates, study drug price discounts, and two study drug discontinuation scenarios (see the Additional Methods and Tables S5-S6 in the online Supporting Information). Sensitivity analyses applying 20–80% discounts on the price of EQW were included to represent the potential impact of price changes due to future patent expiration or additional price negotiations. Seven subgroup analyses were conducted for each country setting.

## 3 Results

### 3.1 Base-case analysis

In both the US and UK analyses, lifetime diabetes medication costs and total costs were significantly higher in the EQW arm (p < 0.001), while costs for medications other than diabetes drugs, outpatient visits, hospitalizations, and complications (‘other costs’) were similar between trial arms (Table 1). Participants in the EQW arm gained significantly more QALYs than those in the placebo group irrespective of the EQ-5D value set used (p < 0.001). The net gain in life-years was approximately 2 months, and the difference in lifetime QALYs was 0.162 for the US (0.151 for the UK).

**Table 1:** Within-trial, post-trial, and lifetime results for US and UK settings (costs in USD and GBP, respectively).

	**Period**	**US**			**UK**		
		**Exenatide**	**Placebo**	**Difference**	**Exenatide**	**Placebo**	**Difference**
Therapy costs (study drug + concomitant diabetes medications)	Within-trial	27,675 (2 0 7)	15,446 (1 7 7)	12,229 (2 7 2)*	3724 (25)	1823 (20)	1901 (32)*
	Post-trial	75,464 (1109)	45,972 (6 7 9)	29,492 (4 3 3)*	9796 (1 3 9)	5143 (73)	4653 (66) *
	Lifetime	103,139 (1131)	61,418 (7 0 2)	41,721 (5 1 3)*	13,520 (1 4 1)	6966 (76)	6554 (73) *
Other costs (other medications, visits, hospitalizations and complications)	Within-trial	15,026 (3 1 0)	15,468 (3 1 9)	−442 (4 4 5)	7151 (1 7 1)	7380 (1 6 9)	−229 (2 4 0)
	Post-trial	98,343 (2117)	98,077 (2154)	266 (1 2 4)	33,654 (7 7 5)	33,621 (7 9 2)	33 (47)
	Lifetime	113,369 (2143)	113,545 (2179)	−176 (4 6 2)	40,805 (7 9 4)	41,001 (8 1 0)	−197 (2 4 5)
Total costs	Within-trial	42,701 (4 0 3)	30,913 (3 9 5)	11,787 (5 6 3)*	10,874 (1 7 7)	9203 (1 7 3)	1671 (2 4 8) *
	Post-trial	173,807 (3012)	144,049 (2687)	29,758 (4 4 0)*	43,450 (8 9 4)	38,765 (8 5 5)	4,686 (76) *
	Lifetime	216,508 (3045)	174,963 (2717)	41,545 (7 1 9)*	54,325 (9 1 3)	47,968 (8 7 2)	6357 (2 6 0) *
QALYs	Within-trial	2.674 (0.013)	2.627 (0.012)	0.047 (0.017)*	2.315 (0.012)	2.272 (0.011)	0.043 (0.016) *
	Post-trial	7.410 (0.108)	7.294 (0.107)	0.115 (0.007)*	7.011 (0.099)	6.903 (0.098)	0.108 (0.006) *
	Lifetime	10.084 (0.109)	9.921 (0.108)	0.162 (0.018)*	9.326 (0.100)	9.175 (0.099)	0.151 (0.017) *
Life years	Within-trial	2.937 (0.065)	2.907 (0.065)	0.030 (0.020)	3.013 (0.038)	2.984 (0.039)	0.029 (0.020)
	Post-trial	9.385 (0.138)	9.245 (0.137)	0.139 (0.008)*	8.877 (0.126)	8.746 (0.125)	0.131 (0.007) *
	Lifetime	12.556 (0.139)	12.386 (0.137)	0.170 (0.022)*	11.890 (0.132)	11.730 (0.130)	0.160 (0.021) *
Cost/QALY	Lifetime			259,223			42,589

Values are means (standard errors).

Abbreviations: GBP, British pounds; QALY, quality-adjusted life-year; USD, US dollars.

* p < 0.05.

**Fig. 1:**
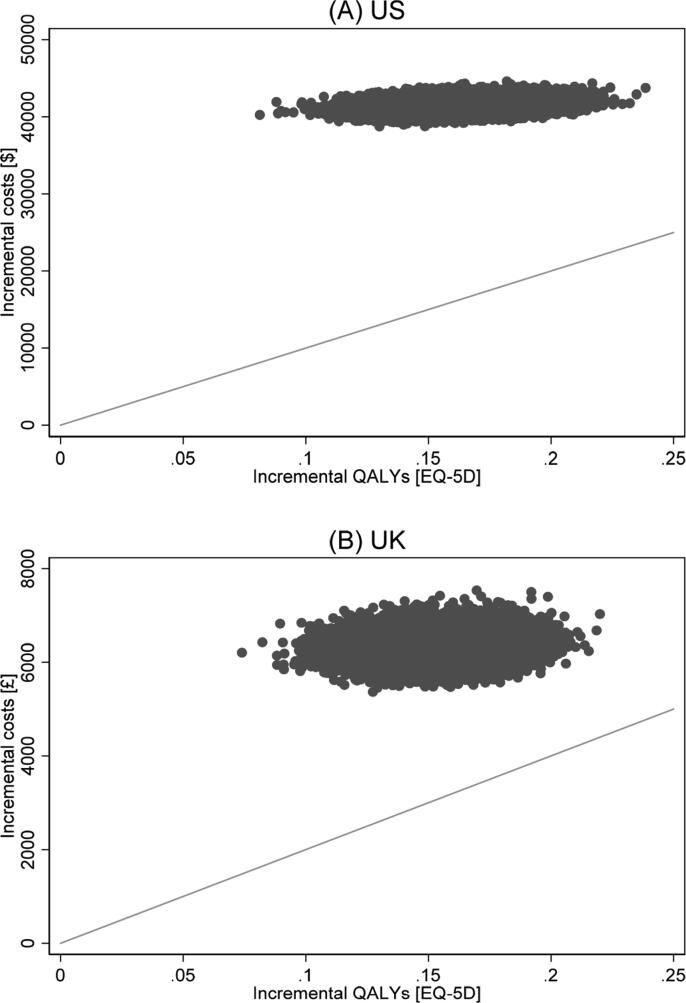
Cost-effectiveness plane showing the distribution of lifetime incremental costs and quality-adjusted life-years (QALYs) in a US setting (**A**) and in a UK setting (**B**). Grey diagonal lines show the value indicating the commonly cited cost-effectiveness thresholds for society’s willingness to pay for a QALY gained (USD 100,000 [**A**] and GBP 20,000 [**B**], respectively).

**Fig. 2:**
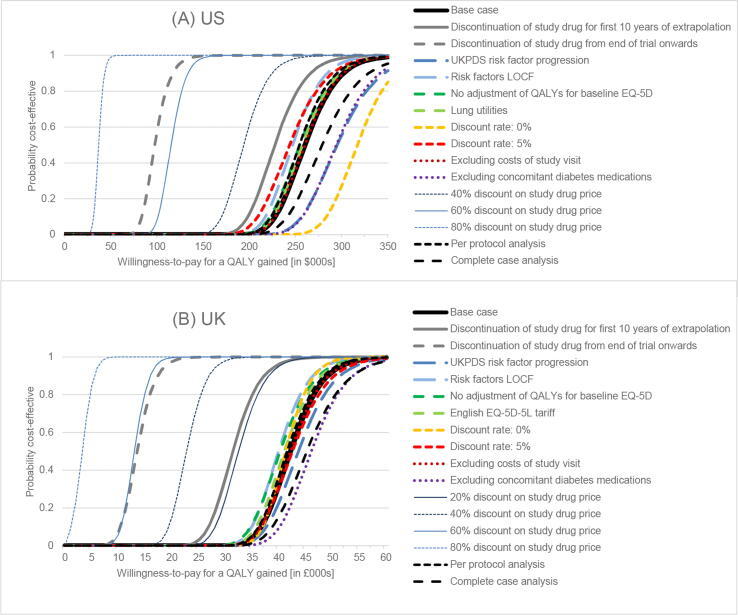
Cost-effectiveness acceptability curves showing how the probability that exenatide is cost-effective compared with standard care varies with the ceiling ratio representing the value that society places on a QALY gained in a US setting (**A**) and in a UK setting (**B**).

Since those randomized to EQW gained 0.162 QALYs (p < 0.001) at an additional cost of USD 41,545 (p < 0.001) per patient, the ICER was estimated as USD 259,223/QALY (95% CI: 211,028 to 324,653), compared with usual care in a US setting. In a UK setting, the estimated net gain was 0.151 QALYs (p < 0.001) at an additional cost of GBP 6357 (p < 0.001), giving an ICER of GBP 42,589/QALY (95% CI: 34,272 to 53,572). The base-case ICERs exceeded the commonly cited cost-effectiveness thresholds of USD 100,000[23] and GBP 20,000 per QALY.[24] The cost-effectiveness planes shown in Fig. 1 show that for both the US and UK analysis, all of the 800 bootstrap iterations for each of the 28 imputed data sets lie in the northeast quadrant, finding EQW to be more costly and more effective than usual care using data from the EXSCEL trial. Fig. 2 shows the cost-effectiveness acceptability curves plotting the probability that the intervention is cost-effective at different willingness-to-pay thresholds.

### 3.2 Sensitivity and subgroup analyses

In most sensitivity analyses, ICERs remained above commonly cited willingness-to-pay thresholds (Fig. 2, Supporting Information). Estimates were in the ranges of USD 34,369–269,571 and GBP 3430–46,560 per QALY gained. In the US setting, only one sensitivity analysis (reducing the EQW price by 80%) and one subgroup (duration of diabetes < 5 years) had an ICER below the threshold of USD 100,000. However, one further sensitivity analysis (reducing the EQW price by 60%) and two further subgroups (patients enrolled in US sites only, age ≥ 65) had ICERs below USD 120,000/QALY. In the UK setting, reducing the EQW price by 40–80% or limiting the analysis to some subgroups (patients enrolled in UK sites only, patients aged ≥ 65 years, duration of diabetes < 5 years) brought the central ICER estimate close to or below the threshold of GBP 20,000/QALY. Subgroups with higher within-trial QALY gains were found to also have higher post-trial QALY gains (Fig. 2, Supporting Information). In discontinuation scenario 1, in which all patients discontinued study drug at the end of the trial (see Supporting Information) and the only source of any subsequent differences in risk was the history of events experienced during the study, the estimated ICERs were USD 98,551/QALY and GBP 14,734/QALY, which represent the minimum ICERs estimated as part of our sensitivity analyses for different adherence assumptions. In discontinuation scenario 2, where study drug therapy continued after the end of the follow-up period but was subject to further discontinuation at the rate observed after the first year of the trial (see Supporting Information), the estimated ICERs were USD 200,076 and GBP 32,799.

Changing the methods used to cost study visits or concomitant medications or to vary utility inputs or the time path trajectories of risk factors in the UKPDS-OM2 had negligible effects on the overall conclusions.

## 4 Discussion

To our knowledge, this is the first study simulating the long-term cost-effectiveness of EQW added to usual care compared to usual care alone. Using data from EXSCEL, a cardiovascular safety outcomes trial, we found that the intervention resulted in statistically significant QALY gains but was more costly than usual care across a lifetime. The base-case ICER exceeded standard cost-effectiveness thresholds. However, ICERs were below or close to the standard thresholds in specific groups, such as patients with a duration of diabetes of < 5 years, aged 65 and older (UK), or those enrolled in US or UK sites only, for whom the main analysis identified a statistically significant treatment effect.[8]

The nominally significant reduction in all-cause mortality observed during the trial and the non-significant changes in risk factors[8] were associated with a QALY gain during the within-trial period and translated into higher QALY gains during the extrapolated period. However, this extended survival resulted in patients in the EQW group necessarily incurring additional costs, increasing total costs in that group.

Costs, effects, and cost-effectiveness differed between the US and UK. The main factor driving the differences was the assumed price of branded EQW (USD 119.70 per dose after applying a 23.1% discount on the wholesale acquisition price vs. GBP 18.94), although there were also marked differences in the costs of concomitant medications and of hospitalizations in the within-trial period, and complications beyond the end of the trial. The US and the UK analyses also used different discount rates and EQ-5D value sets. Further research would be required to assess cost-effectiveness in other countries, particularly in countries where clinical practice and the prices of EQW as well as other healthcare resources differ from those in the US or the UK. QALY gains were larger in the subgroups of patients enrolled in US (n = 3164) or UK sites (n = 347), which increased the probability of EQW being cost-effective. However, while additional subgroup analyses suggested that EQW might be cost-effective for patients who have had diabetes for < 5 years, or those aged ≥ 65 years, it might not be equitable to determine a patient’s eligibility to receive treatment based on their age or an earlier diabetes diagnosis.

The clinical effectiveness from the EXSCEL trial was similar to other trials of cardiovascular outcomes of GLP-1 RAs.[25], [26] Previous cost-effectiveness analyses compared EQW to an active comparator, such as other GLP-1 RAs including dulaglutide, liraglutide, and lixisenatide,[10], [12] insulin glargine,[11], [13], [27] or exenatide twice-daily.[13] Results suggested that EQW had at least a 75% probability of being cost-effective at a threshold of GBP 20,000 in the UK perspective,[10], [11] or 56% at a threshold of USD 50,000 in the US perspective.[27] However, all of these studies used efficacy data from trials that had much smaller sample sizes. Some studies had a shorter follow-up than EXSCEL,[10] or assumed that patients switched treatment completely during the extrapolated period.[11] Furthermore, the EXSCEL study design differed from previous studies that specified an active comparator. EXSCEL was set up as a pragmatic trial to assess the effect of EQW vs. placebo added to existing standard care regimens that could include any class of diabetes medication other than GLP-1 RAs. This reflects the licensed indication for EQW. Further research would be required to identify which of the GLP-1 RAs with favorable risk-benefit balances represent the best value-for-money, individualized to patients’ needs.

This study has several strengths and limitations. This cost-effectiveness analysis was based on patient-level data for 14,752 patients followed up to 6.7 years, which makes it one of the largest samples for a within-trial economic evaluation of GLP-1 RA use in patients with established type 2 diabetes.[28] Trial outcomes were extrapolated using the UKPDS-OM2,[14] which uses data from a population of UK patients recruited between 1977 and 1991 and followed up until 2007. This model has been validated in different populations, including Italy and Netherlands.[28] In a validation study using EXSCEL data, the model gave event rates that were similar to those observed but overestimated numbers of cardiovascular deaths and did not predict the reduction in all-cause mortality seen with EQW.[29]

Although the analysis takes full account of uncertainty around within-trial costs and QALYs, the standard errors around costs and QALYs in the extrapolated period consider only parameter uncertainty around the UKPDS-OM2 risk equations and ignore model uncertainty, uncertainty around risk factor values at the end of the trial, and uncertainty around costs and quality of life before/after complications.[30] Consequently, we may have underestimated the uncertainty around the results.

In particular, the uncertainty modelled in the two discontinuation cases examined in sensitivity analyses may be underestimated. If all patients discontinue active therapy at the end of trial follow-up (discontinuation scenario 1), ICERs were lower than other scenarios, as small benefits continued to accrue from nominally lower within-trial event rates in the EQW trial arm with no additional treatment cost. However, it might be considered clinically implausible to stop therapy at an arbitrary time-point, after an average of 3.3 years on therapy. Discontinuation scenario 2, where discontinuation of the study drug was modelled during the first 10 years of the simulated period at the rate observed after the first year of the trial, may have underestimated the ‘true’ uncertainty. Data available to model this discontinuation case were limited, and various simplifying assumptions were required; for example, alternative treatments and cost inputs were based on averages from previous studies and did not take into account variation in disease progression and treatment escalation (see Supporting Information).

Most other sensitivity analyses conducted showed the conclusions to be robust to changes in the assumptions and methods used. However, since the results were sensitive to the price of EQW, cost-effectiveness should be evaluated in the context of negotiated prices that some payers may be able to attain. Results varied between subgroups; however, some of these results may need to be interpreted cautiously since some subgroups had small samples. Additionally, there was some evidence that treating patients sooner after their initial diabetes diagnosis might improve the cost-effectiveness of the intervention due to the statistically significant reduction in major adverse cardiovascular events (hazard ratio: 0.70, 95% CI: 0.50 to 0.97).[8]

In summary, this study provides estimates of the lifetime healthcare costs and QALYs with EQW treatment. Adding branded EQW to usual care was associated with significantly greater QALY gains and additional costs compared with usual care alone, and the ICERs estimated in this specific trial population were found to exceed standard cost-effectiveness thresholds. The results appeared robust across a range of modelling assumptions. However, results for prespecified subgroups of the trial population suggested that the intervention could be cost-effective if targeted to specific groups, as well as when the price of EQW is generously discounted. It would be of interest to determine whether similar subgroup effects are found in evaluations of other GLP-1 RAs. Finally, our results are based on the results of a single trial, albeit the largest trial to date of EQW in type 2 diabetes, and a full assessment of cost-effectiveness could benefit from supplementing these trial data with information from other trials and observational studies.

## Declaration of Competing Interest

The authors declare that they have no known competing financial interests or personal relationships that could have appeared to influence the work reported in this paper.
