# Supplementary material for: Lifetime cost-effectiveness simulation of once-weekly exenatide in type 2 diabetes: A cost-utility analysis based on the EXSCEL trial
Source: Diabetes Res Clin Pract. 2022 Jan;183:109152. doi: 10.1016/j.diabres.2021.109152 (PMC8844554; doi:10.1016/j.diabres.2021.109152)
Supplement: Supplementary Data 1 [file mmc1.docx]

**Lifetime cost-effectiveness simulation of once-weekly exenatide in type 2 diabetes: a cost-utility analysis based on the EXSCEL trial**

**Becker F, Dakin HA, Reed SD, et al., *Diabetes Research and Clinical Practice***

**Supporting Information**

**Additional methods**

We used the UKPDS outcomes model version 2 (UKPDS-OM2)^1^ to extrapolate costs and outcomes beyond the trial period. Data observed on cardiovascular risk factors and events for each individual trial participant at the end of study follow-up were entered into the UKPDS-OM2. Cardiovascular risk factors included:

- Age
- Duration of diabetes
- Lipoproteins (high-density [HDL], low-density [LDL])
- Blood pressure
- Body mass index (BMI)
- Glycated haemoglobin (HbA1c)
- Haemoglobin
- Heart rate
- White blood cell (WBC) count
- Estimated glomerular filtration rate (eGFR)

Cardiovascular event data were captured as history of ischaemic heart disease (IHD), heart failure, amputation, blindness, renal failure, stroke, myocardial infarction (MI), ulcer, and presence of atrial fibrillation, albuminuria, and peripheral vascular disease (PVD).

1. *Preparing the data ready for imputation and UKPDS-OM2*

Assumptions and data processing steps:

- The ethnicity categories obtained during the trial were reconciled to the three ethnicity categories used in the UKPDS-OM2:
  - 1=‘white and other’ (i.e. including Aboriginal, Asian [Oriental], Hispanic, American Indian, Alaska native, Maori, Hawaiian and other Pacific Islander);
  - 2=‘Black’;
  - 3=‘Asian’ which included “Asian (other)” in order to focus on Indian patients, excluding ‘Asian (Oriental)’; Oriental patients were included in the category of ‘white and other’.
- We assumed that smoking status did not change between date of randomization and end of study date, since no data on smoking status beyond baseline was available. We also assumed that height, sex and ethnicity did not change during the study.
- We obtained data on continuous risk factors (e.g. blood pressure, cholesterol) at baseline (i.e. at the pre-randomisation visit), at the trial termination visit and estimated average values for each year of the trial (e.g. the year 2 value is the average of the values at the 18-month follow-up, the 2-year follow-up and any other follow-ups during that year).
- Extreme outliers (i.e. clinically implausible values) were identified before any imputation to prevent the outcomes model or the imputation models to give extreme predictions for the patients with erroneous values.
  - - Implausible values were defined as those that are outside the ranges set by the UKPDS-OM2:
      - HDL: 0.01-5 mmol/L
      - LDL: 0.01-10 mmol/L
      - Systolic blood pressure: 50-300 mmHg
      - HbA1c: 3-20%
      - Haemoglobin: 5-20 g/dL
      - Heart rate: 40-200 bpm
      - eGFR: 0.1-200 ml/min/1.73m^2^
      - Weight: 10-300 kg
      - Age: 1-200 years
      - Duration of diabetes: 0-1000 years (max = age)
    - Any risk factor values outside these ranges were considered to be medically implausible and were deleted and imputed in the same way as missing values. If the risk factor was within the range specified in the outcomes model, we assumed that the value was correct.
- Data on 11 events (i.e. IHD, heart failure, amputation, blindness, renal failure, stroke, MI, ulcer, atrial fibrillation, albuminuria, PVD) were collected as history of events at baseline and as date of event during the follow-up period.
  - Atrial fibrillation, PVD, and albuminuria were treated in the same way as events.
  - Trial case-report forms (CRFs) recorded whether or not patients had a history of foot ulcers at baseline, but did not record all such ulcers at follow-up times, recording only the date at which gangrene arose. When identifying each patient's history of ulcers, we therefore used the baseline data on ulcers and updated it with the date of gangrene development.
  - No information for history of renal failure were collected at baseline. Since patients with end-stage renal disease or eGFR <30 mL/min/1.73 m² were excluded from the trial, we assumed that no patients had renal failure at baseline.
  - Data on clinical events were assumed to be complete if no event was recorded during the trial. In other words, we assumed that a patient who had no record of a history of MI at baseline and had no dates of subsequent MI events during the follow-up period had not suffered an MI by the end of the trial.
  - The UKPDS variables for events were coded as blank if no baseline history was reported and no event during the follow-up period was recorded. ‘1’ indicated a baseline history or an event during the trial follow-up period.
  - Events occurring after the patient’s end of study/trial termination date were excluded from the analysis.
- Imputation of baseline values
  - Following best practice,^2^ missing baseline values were imputed deterministically rather than using multiple imputation to ensure that baseline values remain independent of treatment allocation and post-baseline outcomes.
  - Missing baseline data on ethnicity and smoking were imputed based on the most common category among trial participants in that patient’s country.
  - For patients with missing data on baseline height, height was imputed based on the mean value in the patient’s country for trial participants of that gender. Missing BMI data were calculated based on the imputed height data for patients with weight data available. If weight data were missing, we imputed BMI as the mean value across observable baseline BMI values and weight was calculated from height and BMI values.
  - We used the age variable used for the clinical analysis.^3^
  - Unconditional mean imputation was applied to all other *baseline* values; we calculated the overall average for baseline EQ-5D and each baseline risk factor across all patients in the trial and applied this to all patients with missing values. This was done to avoid introducing any baseline imbalance between groups.^2^

*Missing data*

We assumed that the dataset included complete data on clinical events and costs during the within-trial period, based on the assumption that patients reported all clinical events and uses of healthcare resources (e.g. hospitalizations) to investigators. Missing data on length of stay were imputed using the methods described previously.^4^

Imputation of risk factor values at last follow-up (e.g. HDL, HbA1c):

- Multiple imputation was done using *mi impute* in Stata, imputing missing values for 2-year and end of trial continuous risk factors (i.e. HDL, LDL, blood pressure, heart rate, HbA1C, eGFR and BMI) using chained equations with truncated regressions for continuous risk factor variables described above.
- We 75 imputations generated sets of imputed values, as this equalled the percentage of patients with missing data on any of the risk factors at either year 2 or trial termination (74.8% = 11,041/14,752).
- The aim of this imputation was to impute end of trial risk factor data for use in the UKPDS-OM2.
- Imputation of risk factors was done separately from imputation of EQ-5D so that we could use the same risk factor data for all analyses regardless of which EQ-5D tariff we were using.
- We omitted people who died during the trial from all imputation models as their data are not extrapolated in the UKPDS-OM2 and their end of trial data are not missing at random.
- The following variables were included in the imputation model:
  - Continuous baseline risk factor values.
  - 2-year (middle of trial) values for continuous risk factors: included as predictors that have missing values (we included whichever value was closest to 2 years out of those available observations that were post-baseline and before year 4).
  - Values for continuous risk factors at each patient’s last follow-up.
  - Age and duration of diabetes were only included at baseline because they would only change 1 year per year for everybody.
  - Gender, ethnicity and smoking were included only at baseline, as they were assumed to remain the same over the follow-up period.
  - Since the UKPDS-OM2 risk equations are based on BMI rather than height and weight, BMI was included in imputation model at baseline, 2 years and end of trial, while height and weight were omitted.
  - We included ethnicity based on the UKPDS categories (black, Indian, and other)
  - Each of the 11 UKPDS events were included as predictors, both at baseline and at end of trial:
    - we did not include the primary composite endpoint as a predictor;
    - we did not include data on events at 2 years as predictors, for simplicity;
    - binary risk factors atrial fibrillation, PVD and albuminuria were treated as events;
    - renal failure was only included at trial termination, because nobody had renal failure at baseline.
  - Time in trial
  - Randomised treatment allocation
  - Geographical region (a proxy for country), divided into Asia-Pacific, Latin America, North America, Eastern Europe and Western Europe.
- We used multiple imputation, but did not propagate the uncertainty around imputed values into the model. Instead, we averaged across all imputed values and extrapolated those averages in the UKPDS-OM2. This:
  - avoided the bias that would have arisen from using complete case analysis when the risk factor data were not missing completely at random;
  - enabled us to impute all post-treatment values for all patients in one step;
  - avoided strong assumptions associated with alternative methods: e.g. last observation carried forward (LOCF) would require assuming that HbA1c has remained unchanged since baseline in the 10% of patients with no post-treatment HbA1c;
  - avoided the need to extrapolate multiple values for each patient using the UKPDS-OM2 and then combine imputation uncertainty with other types of uncertainty after the model had finished.

*EQ-5D*

- We used multiple imputation to impute missing EQ-5D values and propagated uncertainty around imputed values into the estimates of within-trial and lifetime QALYs using Rubin’s rule.^2^
- We imputed the EQ-5D index (not questionnaire responses) since there was very little item nonresponse.
- Imputations on the UK tariff index values were done separately from the imputation of the US tariff index values.
  - The sensitivity analysis using the Devlin EQ-5D-5L tariff for the UK^5^ was a separate third imputation run.
- We did not use multiple imputation to impute baseline EQ-5D since it is recommended that missing baseline values are imputed deterministically in a way that does not depend on treatment allocation or post-baseline outcomes.^2^ Instead, the overall average baseline EQ-5D was assigned to any patients with missing baseline utility.
- We included EQ-5D at three time points in the imputation model: baseline, trial termination and 2 years.
  - - “Baseline” included only screening/randomisation value (complete data after mean imputation);
    - end of trial comprised utility at the trial termination visit or the patient's last follow-up (e.g. trial discontinuation visit) if they were censored/lost before trial termination (this was treated as missing for the patients who died);
    - 2-year values included whichever value was closest to 2 years out of those available observations that were post-baseline and before year 4.
  - In doing so, we focused on capturing enough EQ-5D measurements to calculate QALYs, while avoiding imputation of up to 12 EQ-5D measurements per patient and allowing for administrative censoring, etc. In particular, we aimed to impute middle of trial data for patients who died or were censored without having any post-baseline EQ-5D measurements; we also wanted to get as accurate as possible an EQ-5D profile over the trial period.
  - Nearly all patients (99%) had baseline EQ-5D data and about 90% had end of trial with very little change in EQ-5D over time.
  - We chose 2 years for the “middle of trial” value because this was the modal follow-up time and just over 50% of the median follow-up time, which meant that it was approximately halfway between randomisation and trial termination for the median patient.
- The number of multiple imputations generated was set to equal 28 as this was the percentage of patients with any missing data on any of the three EQ-5D variables included in the imputation model (4033/14752 = 27.3%).
- Covariates within the imputation model comprised:
  - randomised treatment allocation;
  - pre-randomisation values of all UKPDS risk factors, including events;
  - a dummy variable for the primary endpoint (i.e. cardiovascular death, non-fatal MI, non-fatal stroke);
  - age at baseline;
  - geographical region (a proxy for country, divided into Asia-Pacific, Latin America, North America, Eastern Europe and Western Europe);
  - we did not include country dummies in the imputation model because these could have introduced convergence issues from small countries and co-linearity with variables such as ethnicity;
  - time in trial (because it might predict trial termination utility);
  - We categorised ethnicity based on the UKPDS categories (Black, Indian, and other);
  - we did not explicitly include death as a covariate;
  - we did not include within-trial costs in the imputation model;
  - baseline BMI was used as a predictor in preference to weight or height.
- Multiple imputation was done using *mi impute* in Stata version 14.2.^6^
- Model specification: *truncreg* to impute EQ-5D utilities, censoring at ll(-0.81) ul(1) for US utilities^7^ and ll(-0.594) ul(1) for the analysis of UK utilities.^8^ The sensitivity analysis using the Devlin EQ-5D-5L tariff used the same range as the UK utilities, because the range for EQ-5D-5L [-0.285; 1] is smaller than the range for EQ 5D-3L [-0.594; 1].^5,8^
- QALYs were calculated for each patient up to the point where each patient was considered to leave the trial and enter the model, across all values that were available after imputation: for some patients, this may have been just imputed values at baseline, 2 years and end of trial, while others may have observed values at up to 12 time points.
  - Assumed linear interpolation between time points (i.e. join the dots, or take the average between two adjacent EQ-5D values and multiplied it by the time interval between them).
  - We assumed that observed measurements were done at exactly the same time point as they were scheduled to be, e.g. a 2-year measurement was assumed to have happened exactly 24 months after randomisation.
  - When using values that were imputed at “2 years”, we assumed that the imputed value was “measured” exactly 2 years after randomisation.
  - Unscheduled EQ-5D measurements were included in QALY calculations, as were measurements >4 years after randomization.
  - The QALY calculation included discounting at the appropriate value.
  - We imputed utilities for the trial termination visit for all patients (regardless of whether they had died or been censored).
  - However, the utilities imputed for the trial termination visit were not used for patients who had died. Instead:
    - For people who died after having one or more observed post-baseline values, we calculated the area under the curve between the observed values and then carried forward the last observed value to the day of death.
    - For people who had no post-baseline values, we used the imputed value at “2 years” and treated this as the utility on the day the patient died when calculating the area under the curve: e.g. if someone had baseline and an imputed 2 year utility and died at 2.75 years, we assumed that the imputed 2-year utility was measured at 2.75 years and that utility changed linearly from baseline up to the imputed “2-year” value and then decreased to 0.

1. *Extrapolation outcomes using UKPDS-OM2*

- We used the in house v2.01b8 version of the UKPDS-OM2 in order to obtain a copy of the BootstrapBoots matrix showing outcomes for each patient in each bootstrap, which facilitated subgroup and sensitivity analyses. This gives results that are equivalent to those generated in version 2.1.
- We extrapolated end of trial data for actual trial participants, as randomised (as opposed to applying modelled treatment effects).
- Outcomes were extrapolated for all patients who had not died during the trial.
- Outcomes were extrapolated for 70 years. In the base-case analyses, we assumed that all risk factors (other than age, duration of diabetes and event history) remained at the values observed at the end of the trial for the rest of each patient’s life. Constant risk factors were used as the risk factor trajectories for UKPDS-OM2 version 2 had not been published and the model applying them was still being finalised. A sensitivity analysis used the unpublished risk factor trajectories.
- To reflect parameter uncertainty, we used 800 bootstraps, i.e. the number needed to give SEs to ±10% accuracy.^9^
- To minimise the Monte Carlo error, we did 1000 loops (i.e. 1000 potential histories for each patient) for each bootstrap, which was found to be sufficient to give stable results (at least for the grand mean across all bootstraps) and so that standard errors did not decrease when increasing the number of loops.
- The discounting start year was identified by rounding the duration of follow-up per patient (in years) down to the nearest whole year.
- The US analysis was discounted at 3% per annum and the UK analysis at 3.5% per annum, with no change of discount rates over time.
- Since no data on WBC had been collected, we assumed that all patients had WBC=6.8 at baseline and at their last observation, based on the Lipids in Diabetes study.^1^
- Discontinuation case 1: This sensitivity analysis modelled immediate discontinuation where all study drug use stopped at the end of the trial. Control group risk factor values observed at the end of the trial were assumed to apply over the complete simulation period for both trial arms. No alternative treatment was modelled; the mean costs for the control group in the last full year of the trial was applied to all individuals.
- Discontinuation case 2: This sensitivity analysis accounted for premature study drug discontinuation during the first 10 years of the simulation assuming a discontinuation rate of 9% per year in the EQW arm. This was based on the average annual premature study drug discontinuation rate that was observed after the first year of the trial (i.e. year 2 onwards).
  - Over the trial period, 44% of participants across both groups discontinued the study medication prior to the end of follow-up (exenatide 43.0%; placebo 45.2%).
  - We assumed that patients who were still on treatment after the first 10 years of the simulation would remain on treatment for life unless they developed renal failure. Given discounting of costs and outcomes, and an average patient age of over 64 years when entering the simulation, limiting the discontinuation analysis to the first 10 years of the simulation was assumed to capture the major impacts on costs and outcomes. The impact of discontinuations after this period was assumed to be negligible since only 22% of patients were assumed to remain on treatment after year 10.
  - This analysis followed an approach used by Clarke et al.^10^ and converged risk factor values to the overall mean values until discontinuation (averaged across the control and exenatide arms) and used control group average risk factor values for all individuals for the year following discontinuation. Previous findings from the literature were used to inform additional assumptions on type of alternative treatment and associated costs for the US^11,12^ and the UK^13^ (see Table S1 for details). Uncertainty around risk factors and outcomes was fully captured, while therapy costs for alternative treatment after discontinuation of exenatide (including the yearly increase in diabetes therapy costs capturing disease progression and treatment escalation) could only be captured by point estimates of average costs.
  - Given an average duration of diabetes of 16 years at the beginning of the simulation, patients were assumed to continue with second-line treatment when discontinuing study medication.
  - Additionally, assuming a constant yearly discontinuation rate of 9% may not be a true reflection of patient adherence to the treatment regimen. Discontinuation was primarily due to patients’ decisions as new drugs and administration devices were introduced to the market, so the true discontinuation rate may be less than 9% per year after the first 1-7 years.
  - This discontinuation analysis therefore reflects a sensitivity analysis, while the base-case analysis assumed lifelong treatment for those patients who remained on study medication over the trial period, which allowed us to fully model uncertainty around the results in the base case.

Table S1: Additional input data for discontinuation case 2

| **Parameter** | **Value** | **Source & justification** |
| --- | --- | --- |
| *US* | | |
| Annual exenatide costs before discontinuation | $3,080 | Trial: composite value including exenatide costs and negative difference in concomitant medications between exenatide and control groups; including 23.1% discount on wholesale acquisition price of branded exenatide |
| Annual therapy costs for additional treatment after discontinuation (initial value) | $1,134 | Difference in diabetes-related outpatient medication costs between age groups 45-64 and 65+ years to capture change in treatment with increasing duration of diabetes^11^ |
| % increase in annual therapy costs for second line treatment after discontinuation | 5% | Bonafede et al.^12^ |
| Upper limit of additional annual therapy costs for second line treatment after discontinuation | $14,580 | Trial; 95% percentile of therapy costs for control group participants (based on data available from last full year for each patient) |
| *UK* | | |
| Annual exenatide costs before discontinuation | £517 | Trial: composite value including exenatide costs and negative difference in concomitant medications between exenatide and control groups |
| Annual therapy costs for additional treatment after discontinuation (initial value) | £187 | Eibich et al.^13^ |
| % increase in annual therapy costs for second line treatment after discontinuation | 20% | Eibich et al.^13^ |
| Upper limit of additional annual therapy costs for second line treatment after discontinuation | £1,605 | Trial; 95% percentile of therapy costs for control group participants (based on data available from last full year for each patient) |

*Costs*

- The annual therapy costs entered into the model were based on the mean annual cost of study medication plus concomitant diabetes medications. They were based on the amount of exenatide that was actually being received at the end of the study (i.e. during the last complete year of the study for each patient) to reflect the level of medication that had given rise to the risk factors being used in the model. The cost of exenatide per patient-year was assumed to equal the cost per dose (USD 119.70 after applying a 23.1% on the US wholesale acquisition price or GBP 18.94^14^) multiplied by the mean number of doses received each year by trial participants who were still alive and receiving treatment at the end of the trial (n=4,172), multiplied by the proportion of surviving patients who were still receiving randomised exenatide treatment at the end of follow-up. This gave the costs shown in Table S2.
- Treatment costs in the standard care arm included only the cost of concomitant diabetes medication; all other treatments and medications were assumed to be included in the costs with and without diabetic events. Patients were assumed to stop exenatide if they developed renal failure, but all other therapy costs were assumed to remain the same and the base-case analysis assumed that patients without renal failure continued to receive exenatide for life.

*Table S2. Annual cost of diabetes medication applied in each year of life extrapolated using the UKPDS-OM2*

|  | **Placebo** | | **Exenatide** | |
| --- | --- | --- | --- | --- |
|  | **US** | **UK** | **US** | **UK** |
| Annual therapy cost prior to renal failure | $4,972.51 | £588.06 | $8,052.12 | £1,105.40 |
| Annual therapy cost following renal failure | $4,972.51 | £588.06 | $4,972.51 | £588.06 |

- The US cost of managing diabetes in patients who have not had diabetic events was based on a estimates by the American Diabetes Association (ADA),^15^ supplemented by data from EXSCEL (Table S3). The ADA’s estimates were adjusted to calculate lifetime costs in line with published EXSCEL within-trial cost calculations reflecting a Medicare perspective.^4^ We included medication and outpatient cost based on EXSCEL resource use data, and excluded the ADA’s estimates for inpatient, emergency department, nursing/residential/hospice/ambulance services, home health, podiatry, and other equipment and supplies assuming that those were due to complications. Within the US analysis, cost estimates for diabetic events published by Ward et al.^16^ were adjusted for inflation (Table S4), updated from 2012 values to 2017 values using the medical care component of the Consumer Price Index published by the US Bureau of Labor Statistics.^17^
- The UK analysis used the costs estimated by Alva et al.^18,19^ by age group and gender, adjusted for inflation. Within the UK analysis, costs were adjusted for health care inflation to bring values up to 2015/16 using the hospital and community health services pay and prices index.^20^

*Utilities*

- During the extrapolated period, the US and UK base-case analyses used the utilities (with and without diabetic events) that were estimated by Alva et al.^18^
- Alternative US-based estimates from a study by Lung et al.^21^ were used in the model in a sensitivity analysis.

*Table S3. Annual mean cost per person in absence of complications, applied in the US analysis (in 2017 prices).*

| **Cost component** | **Annual mean cost per person (in USD)** | **Source** |
| --- | --- | --- |
| Physician office visits | 2,100 | ADA^15^* |
| Hospital outpatient visits | 648 | EXSCEL^4^† |
| Diabetes supplies | 151 | ADA^15^‡ |
| Prescription medications | 985 | EXSCEL^4^§ |
| *Total* | *3,885* |  |

*Total of USD 51,882 million incurred by 24.7 million people with diagnosed diabetes.

†Mean cost in placebo group over average follow-up duration of 3.3 years: USD 2,139.

‡Total of USD 3,723 million incurred by 24.7 million people with diagnosed diabetes.

§Mean cost in placebo group over average follow-up duration of 3.3 years: USD 3,252; excluding insulin and other anti-diabetes agents, since already included in EXSCEL concomitant diabetes medications that were captured elsewhere in the UKPDS-OM2.

*Table S4. Costs and utilities with and without cardiovascular events applied in the US analysis. Costs and utilities were assumed to not vary with age or gender*

|  | **At time of event** | | | | **In subsequent years** | |  |
| --- | --- | --- | --- | --- | --- | --- | --- |
|  | **Fatal cost** | **Non-fatal cost*** | **Utility decrement: base-case analysis^18^** | **Utility decrement: sensitivity analysis^21^** | **Annual cost*** | **Utility decrement: base-case analysis^18^** | **Utility decrement: sensitivity analysis^21^** |
| IHD | 24,617.18* | 24,617.18 | 0.000 | -0.090 | 2,189.63 | 0.000 | -0.090 |
| MI | 64,912.50* | 64,912.50 | -0.065 | -0.060 | 2,189.63 | 0.000 | -0.060 |
| Heart failure | 0.00† | 27,322.01 | -0.101 | -0.110 | 2,189.63 | -0.101 | -0.110 |
| Stroke | 48,437.41* | 48,437.41 | -0.165 | -0.220 | 17,872.36 | -0.165 | -0.220 |
| Amputation | 0.00† | 10,397.27 | -0.172 | -0.250 | 0.00 | -0.172 | -0.250 |
| Blindness |  | 3,291.34 | 0.000 | -0.280 | 3,291.34 | 0.000 | -0.280 |
| Renal failure | *0.00*† | *82,472.05* | *-0.330* | *-0.330* | *82,472.05* | *-0.330* | *-0.330* |
| Ulcer |  | *2,469.08* | *-0.210* | *-0.104* | *2,469.08* | *-0.210* | *-0.104* |
|  |  |  |  |  |  |  |  |
| Utility in the absence of complications | 0.807(3) | *( 0 to 1 )* |  | 0.810 |  |  |  |
| Cost in the absence of complications | 3.884,85‡ |  |  | 3.884,85‡ |  |  |  |

*Ward et al.^16^ adjusted for inflation to 2017 US dollars. Since this study did not differentiate between fatal and non-fatal events, the cost in the year of the event was assumed to be the same.

†Assumed to be zero, following the default assumption in the UKPDS-OM2.

‡American Diabetes Association^15^ and EXSCEL data in 2017 US dollars (see Table S3).

1. *Analysing and presenting UKPDS model output*

- We calculated lifetime costs and QALYs by drawing 800 bootstraps from the EXSCEL within-trial data and generating 800 bootstrapped estimates of outcomes in the period extrapolated using the UKPDS-OM2. The UKPDS-OM2 contains 5000 sets of bootstrapped risk equation parameters that were previously bootstrapped from the UKPDS trial sample.^1,22^ We used the UKPDS-OM2 to simulate 800 bootstrapped estimates of outcomes for each patient using different estimates of risk equation parameters using bootstraps drawn from the UKPDS trial sample; each of these 800 bootstraps were used to predict lifetime costs and QALYs for each of the EXSCEL participants.
- Bootstrapping the within-trial period:
- We used data on the discounted within-trial costs for each patient up to the end of trial date.
- We drew 800 bootstraps for each of the 28 imputed within-trial EXSCEL data sets (taken from the imputation of EQ-5D utilities), stratifying by treatment group as well as imputation number.
  - For the subgroup analyses and complete case analysis, we dropped patients who were not in that subgroup before bootstrapping, so that we had the same patient numbers in each bootstrap.
- Mean within-trial costs and life years in the exenatide and placebo groups were calculated for each EXSCEL bootstrap in each imputed dataset without adjusting for any covariates.
- Ordinary least squares regression (OLS) was used to adjust for baseline EQ-5D utility when estimating mean within-trial QALYs in the exenatide and placebo groups for each EXSCEL bootstrap in each imputed dataset. This was done to eliminate any bias that may have resulted from between-group imbalance in baseline utility.^23^
- OLS regression was done for QALYs, life years, different categories of US and UK costs, and different sensitivity and subgroup analyses, using the same bootstraps. We allowed for correlations between costs and QALYs/life years by using the same bootstraps for both. Running all of the sensitivity analyses on the same bootstraps avoided chance differences between sensitivity analyses.
- Costs, life years and QALYs accrued in the extrapolated period in each UKPDS bootstrap of each study arm were multiplied by the proportion of patients in that treatment arm who were still alive at the end of the study to allow for within-trial mortality.
  - The proportion of patients who were still alive at the end of the study was fixed across all bootstraps. This ensured consistent results and reflects the fact that we ignored sampling uncertainty when analysing extrapolated outputs.
- We duplicated each of the 800 UKPDS bootstraps of each study arm 28 times to give a total of 22,400 estimates of post-trial costs and effects: one for each of the 800 bootstraps of each of 28 imputations that we had for within-trial data.
- We added mean within-trial outcomes for each study arm in each of the 22,400 within-trial bootstraps to the mean outcomes for one of the UKPDS bootstraps of the extrapolated period to give total lifetime costs, QALYs and life years.
  - This assumed no correlation between the extrapolated period and the within-trial period, because the UKPDS bootstraps represent only the impact of parameter uncertainty between bootstraps drawn from the UKPDS trial, while the bootstrapped within-trial outcomes represent only sampling uncertainty between EXSCEL participants.
- Differences between the EQW and placebo groups were calculated for each of the 22,400 bootstraps.
- We averaged across all 22,400 bootstraps to get point estimates.
  - By contrast, the default within the UKPDS is to base point estimates on the outcomes predicted in bootstrap 0 (i.e. the outcomes when using the mean coefficients for each risk equation). However, unlike our approach, the UKPDS default is not compatible with the bootstrap approach we used to combine the within-trial and extrapolated uncertainty, did not allow for non-linearity and would also have required a larger number of loops and therefore increased computation time.
  - Subgroup analyses and sensitivity analyses that focussed on a subset of patients (e.g. complete case analysis) were conducted by taking the average across the relevant patient group within each bootstrap.
- Standard errors (SEs) around total and incremental outcomes were then calculated in Microsoft Excel using Rubin’s rule^2^ to combine results across the 28 imputed datasets, by adding the bias-adjusted variance between the 28 imputations to the variance within each imputed dataset.
  - We calculated the variance within each imputed dataset by taking the standard deviation across the 800 bootstraps. This is extremely similar to the standard way that the UKPDS-OM2 presents uncertainty around model results and to the way that it is used in practice, except that the outcomes model normally uses the percentile method to obtain confidence intervals rather than assuming a normal distribution. However, this approach included only parameter uncertainty around the outcomes in the extrapolated period and ignored the uncertainty around the risk factor data coming from the EXSCEL sample.^24^ It underestimated uncertainty by assuming that the EXSCEL sample represented the entire population of interest during the extrapolated period (although we allowed for sampling uncertainty fully during the within-trial period).
- Scatter graphs were generated by plotting the incremental costs and QALYs across all 22,400 bootstraps.
- Cost-effectiveness acceptability curves (CEACs) were generated by calculating net monetary benefit (NMB) for each bootstrap at a range of ceiling ratios and plotting the proportion of bootstraps where the incremental NMB was positive.
- We did not conduct a multilevel analysis adjusting lifetime outcomes and costs for country random effects, because this was not feasible using the methods applied.
- Net benefits were presented at a USD 100,000/QALY ceiling ratio for the US and a GBP 20,000/QALY ceiling ratio for the UK.

*Table S5. Sensitivity and subgroup analyses - US*

| **US analysis** | **N** | **Incremental cost (SE) [USD]** | **Incremental QALYs (SE)** | **Cost/QALY (Probability EQW is cost-effective at USD 100,000 threshold)** |
| --- | --- | --- | --- | --- |
| *Base case* | *E: 7,356; P: 7,396* | *41,545 (719)* | *0.162 (0.018)* | *259,223 (0%)* |
| *Sensitivity analyses* |  |  |  |  |
| Using risk factor progression models from UKPDS OM 2.01b8 | E: 7,356; P: 7,396 | 39,463 (680) | 0.150 (0.018) | 267,544 (0%) |
| Last observation carried forward for risk factors (including baseline values) in lieu of multiple imputation (MI) | E: 7,356; P: 7,396 | 41,353 (720) | 0.171 (0.018) | 244,897 (0%) |
| No adjustment for baseline utility when calculating within-trial QALYs | E: 7,356; P: 7,396 | 41,545 (719) | 0.166 (0.019) | 253,996 (0%) |
| Using estimates of utilities from Lung et al.^21^ | E: 7,356; P: 7,396 | 41,545 (719) | 0.164 (0.018) | 257,164 (0%) |
| Discount rate: 0% | E: 7,356; P: 7,396 | 61,417 (1,062) | 0.230 (0.022) | 269,571 (0%) |
| Discount rate: 5% | E: 7,356; P: 7,396 | 30,879 (619) | 0.134 (0.017) | 233,880 (0%) |
| Applying 40% discount to US EQW price* | E: 7,356; P: 7,396 | 30,846 (655) | 0.162 (0.018) | 192,439 (0%) |
| Applying 60% discount to US EQW price* | E: 7,356; P: 7,396 | 18,184 (599) | 0.162 (0.018) | 113,404 (13.1%) |
| Applying 80% discount to US EQW price* | E: 7,356; P: 7,396 | 5,522 (571) | 0.162 (0.018) | 34,369 (100%) |
| Discontinuation scenario 1: all patients stop study drug immediately after end of trial | E: 7,356; P: 7,396 | 12,805 (579) | 0.132 (0.018) | 98,551 (59%) |
| Discontinuation scenario 2: a proportion of patients stop study drug during first 10 years of simulation | E: 7,356; P: 7,396 | 30,080 (598) | 0.152 (0.018) | 200,076 (0%) |
| Excluding costs of study visit | E: 7,356; P: 7,396 | 45,676 (724) | 0.162 (0.018) | 285,004 (0%) |
| Excluding concomitant diabetic medications from the therapy costs used in the model | E: 7,356; P: 7,396 | 47,020 (766) | 0.162 (0.018) | 293,410 (0%) |
| Complete case† | E: 6,801; P: 6,786 | 41,927 (738) | 0.152 (0.019) | 277,622 (0%) |
| Per protocol analysis | E: 7,263; P: 7,302 | 41,393 (719) | 0.165 (0.018) | 253,427 (0%) |
| *Subgroup analyses* |  |  |  |  |
| Patients enrolled in US sites only | E: 1,567; P: 1,597 | 41,673 (1,500) | 0.416 (0.046) | 101,439 (49%) |
| Age <65yrs | E: 4,392; P: 4,421 | 47,161 (875) | 0.085 (0.024) | 606,429 (0%) |
| Age ≥65yrs | E: 2,964; P: 2,975 | 33,316 (1,024) | 0.283 (0.028) | 118,783 (3%) |
| Duration of diabetes <5yrs | E: 1,032; P: 980 | 44,414 (1,259) | 0.452 (0.051) | 99,491 (56%) |
| Duration of diabetes ≥5yrs & <15yrs | E: 3,637; P: 3,682 | 44,479 (891) | -0.004 (0.026) | Dominated |
| Duration of diabetes ≥15yrs | E: 2,687; P: 2,734 | 36,176 (1,193) | 0.240 (0.028) | 152,570 (0%) |

SE = Standard errors; E = exenatide; P = placebo. *Sensitivity analyses applying a 40-80% discount on exenatide price are based on the full wholesale acquisition costs and do not apply the discount on the base-case price which already includes a 23.1% discount. ^†^Including complete cases only, in order to identify potential differences in results between the MI approach and the complete case analysis, and to assess the validity of the MI results. For this purpose, complete cases were defined by EQ-5D values available at baseline and at one or more post-baseline time points.

*Table S6. Sensitivity and subgroup analyses - UK*

| **UK analysis** | **N** | **Incremental cost (SE) [GBP]** | **Incremental QALYs (SE)** | **Cost/QALY (Probability EQW is cost-effective at GBP 20,000 threshold)** |
| --- | --- | --- | --- | --- |
| *Base case* | *E: 7,356; P: 7,396* | *6,357 (260)* | *0.151 (0.017)* | *42,589 (0%)* |
| *Sensitivity analyses* |  |  |  |  |
| Using risk factor progression models from UKPDS OM 2.01b8 | E: 7,356; P: 7,396 | 6,071 (257) | 0.139 (0.016) | 44,204 (0%) |
| Last observation carried forward for risk factors (including baseline values) in lieu of multiple imputation (MI) | E: 7,356; P: 7,396 | 6,328 (260) | 0.159 (0.017) | 40,360 (0%) |
| No adjustment for baseline utility when calculating within-trial QALYs | E: 7,356; P: 7,396 | 6,357 (260) | 0.158 (0.018) | 40,716 (0%) |
| Using the English EQ-5D-5L tariff ^5^ to value EQ-5D-5L questionnaires, while continuing to use the Dolan tariff ^8^ for EQ-5D-3L responses | E: 7,356; P: 7,396 | 6,357 (260) | 0.155 (0.018) | 41,491 (0%) |
| Discount rate: 0% | E: 7,356; P: 7,396 | 9,340 (311) | 0.226 (0.021) | 41,713 (0%) |
| Discount rate: 5% | E: 7,356; P: 7,396 | 5,557 (249) | 0.131 (0.016) | 43,015 (0%) |
| Applying 20% discount to UK EQW price | E: 7,356; P: 7,396 | 4,897 (257) | 0.151 (0.017) | 32,867 (0%) |
| Applying 40% discount to UK EQW price | E: 7,356; P: 7,396 | 3,436 (255) | 0.151 (0.017) | 23,055 (15%) |
| Applying 60% discount to UK EQW price | E: 7,356; P: 7,396 | 1,975 (253) | 0.151 (0.017) | 13,243 (100%) |
| Applying 80% discount to UK EQW price | E: 7,356; P: 7,396 | 514 (249) | 0.151 (0.017) | 3,430 (100%) |
| Discontinuation scenario 1: all patients stop study drug immediately after end of trial | E: 7,356; P: 7,396 | 1,788 (252) | 0.123 (0.017) | 14,734 (96%) |
| Discontinuation scenario 2: a proportion of patients stop study drug during first 10 years of simulation | E: 7,356; P: 7,396 | 4,599 (253) | 0.142 (0.017) | 32,799 (0%) |
| Excluding costs of study visit | E: 7,356; P: 7,396 | 6,352 (260) | 0.151 (0.017) | 42,642 (0%) |
| Excluding concomitant diabetic medications from the therapy costs used in the model | E: 7,356; P: 7,396 | 6,935 (257) | 0.151 (0.017) | 46,560 (0%) |
| Complete case* | E: 6,801; P: 6,786 | 6,383 (273) | 0.142 (0.017) | 45,691 (0%) |
| Per protocol analysis | E: 7,263; P: 7,302 | 6,348 (263) | 0.153 (0.017) | 41,960 (0%) |
| *Subgroup analyses* |  |  |  |  |
| Patients enrolled in UK sites only | E: 177; P: 170 | 11,787 (1,888) | 0.595 (0.090) | 20,284 (57%) |
| Age <65yrs | E: 4,392; P: 4,421 | 7,208 (318) | 0.081 (0.022) | 97,295 (0%) |
| Age ≥65yrs | E: 2,964; P: 2,975 | 5,102 (420) | 0.261 (0.026) | 19,742 (57%) |
| Duration of diabetes <5yrs | E: 1,032; P: 980 | 5,599 (526) | 0.410 (0.047) | 13,807 (99%) |
| Duration of diabetes ≥5yrs & <15yrs | E: 3,637; P: 3,682 | 7,238 (341) | -0.001 (0.024) | Dominated |
| Duration of diabetes ≥15yrs | E: 2,687; P: 2,734 | 5,453 (484) | 0.224 (0.026) | 24,653 (7%) |
| Patients with CVD history at baseline | E: 5,394; P: 5,388 | 5,666 (325) | 0.161 (0.018) | 35,522 (0%) |

SE = Standard errors; E = exenatide; P = placebo. *Including complete cases only, in order to identify potential differences in results between the MI approach and the complete case analysis, and to assess the validity of the MI results. For this purpose, complete cases were defined by EQ-5D values available at baseline and at one or more post-baseline time points.

**References**

1. Hayes AJ, Leal J, Gray AM, Holman RR, Clarke PM. UKPDS outcomes model 2: a new version of a model to simulate lifetime health outcomes of patients with type 2 diabetes mellitus using data from the 30 year United Kingdom Prospective Diabetes Study: UKPDS 82. Diabetologia. 2013;56:1925-33.

2. White IR, Royston P, Wood AM. Multiple imputation using chained equations: Issues and guidance for practice. Stat Med. 2011;30:377-99.

3. Holman RR, Bethel MA, Mentz RJ, Thompson VP, Lokhnygina Y, Buse JB, Chan JC, Choi J, Gustavson SM, Iqbal N, Maggioni AP, Marso SP, Ohman P, Pagidipati NJ, Poulter N, Ramachandran A, Zinman B, Hernandez AF; EXSCEL Study Group. Effects of once-weekly exenatide on cardiovascular outcomes in type 2 diabetes. N Engl J Med. 2017;377:1228-39.

4. Reed SD, Li Y, Dakin HA, Becker F, Leal J, Gustavson SM, Kartman B, Wittbrodt E, Mentz RJ, Pagidipati NJ, Bethel MA, Gray AM, Holman RR, Hernandez AF; EXSCEL Study Group. Within-trial evaluation of medical resources, costs, and quality of life among patients with type 2 diabetes participating in the Exenatide Study of Cardiovascular Event Lowering (EXSCEL). Diabetes Care. 2020;43:374-81.

5. Devlin NJ, Shah KK, Feng Y, Mulhern B, van Hout B. Valuing health-related quality of life: An EQ-5D-5L value set for England. Health Econ 2018;27:7-22.

6. StataCorp. Stata Statistical Software: Release 14: College Station, TX: StataCorp LP, 2015.

7. Shaw JW, Pickard AS, Yu S, Chen S, Iannacchione VG, Johnson JA, Coons SJ. A median model for predicting United States population‐based EQ‐5D health state preferences. Value Health. 2010;13:278-88.

8. Dolan P. Modeling valuations for EuroQol health states. Med Care. 1997;35:1095-108.

9. O'Hagan A, Stevenson M, Madan J. Monte Carlo probabilistic sensitivity analysis for patient level simulation models: efficient estimation of mean and variance using ANOVA. Health Econ .2007;16:1009-23.

10. Clarke P, Gray A, Briggs A, Stevens R, Matthews D, Holman R. Cost-utility analyses of intensive blood glucose and tight blood pressure control in type 2 diabetes (UKPDS 72). Diabetologia 2005;48:868-77.

11. American Diabetes Association. Economic costs of diabetes in the U.S. in 2012. Diabetes Care. 2013;36:1033-46.

12. Bonafede M, Chandran A, DiMario S, Saltiel-Berzin R, Saliu D. Medication usage, treatment intensification, and medical cost in patients with type 2 diabetes: a retrospective database study. BMJ Open Diabetes Res Care. 2016;4:e000189.

13. Eibich P, Green A, Hattersley AT, Jennison C, Lonergan M, Pearson ER, Gray AM. Costs and treatment pathways for type 2 diabetes in the UK: a mastermind cohort study. Diabetes Ther. 2017;8:1031-45.

14. British National Formulary. https://www.medicinescomplete.com/#/content/bnf/_853586898?hspl=Exenatide&hspl=price#DMD28426011000001108 (accessed 25 Sept 2017).

15. American Diabetes Association. Economic costs of diabetes in the US in 2017. Diabetes Care. 2018;41:917.

16. Ward A, Alvarez P, Vo L, Martin S. Direct medical costs of complications of diabetes in the United States: estimates for event-year and annual state costs (USD 2012). J Med Econ. 2014;17:176-83.

17. US Bureau of Labor Statistics. https://data.bls.gov/cgi-bin/surveymost?cu (accessed 25th Sept 2018).

18. Alva M, Gray A, Mihaylova B, Clarke P. The effect of diabetes complications on health-related quality of life: the importance of longitudinal data to address patient heterogeneity. Health Econ. 2014;23:487-500.

19. Alva ML, Gray A, Mihaylova B, Leal J, Holman RR. The impact of diabetes-related complications on healthcare costs: new results from the UKPDS (UKPDS 84). Diabet Med. 2015;32:459-66.

20. Curtis L, Burns A. Unit costs of health and social care 2015. Canterbury, England: Personal Social Services Research Unit, University of Kent, 2015.

21. Lung TW, Hayes AJ, Hayen A, Farmer A, Clarke PM. A meta-analysis of health state valuations for people with diabetes: explaining the variation across methods and implications for economic evaluation. Qual Life Res 2011;20:1669-78.

22. Clarke PM, Gray AM, Briggs A, Farmer AJ, Fenn P, Stevens RJ, Matthews DR, Stratton IM, Holman RR; UKPDS Group. A model to estimate the lifetime health outcomes of patients with type 2 diabetes: the United Kingdom Prospective Diabetes Study (UKPDS) Outcomes Model (UKPDS no. 68). Diabetologia. 2004;47:1747-59.

23. Manca A, Hawkins N, Sculpher MJ. Estimating mean QALYs in trial-based cost-effectiveness analysis: the importance of controlling for baseline utility. Health Econ 2005;14:487-96.

24. Dakin HA, Leal J, Briggs A, Clarke P, Holman RR, Gray A. Accurately reflecting uncertainty when using patient-level simulation models to extrapolate clinical trial data. Med Decis Making. 2020;40:460-73.
